# Imaging blood–brain barrier dysfunction in drug‐resistant epilepsy: A multi‐center feasibility study

**DOI:** 10.1111/epi.18145

**Published:** 2024-11-06

**Authors:** Nir Cafri, Sheida Mirloo, Daniel Zarhin, Lyna Kamintsky, Yonatan Serlin, Laith Alhadeed, Ilan Goldberg, Mark A. Maclean, Ben Whatley, Ilia Urman, Colin P. Doherty, Chris Greene, Claire Behan, Declan Brennan, Matthew Campbell, Chris Bowen, Gal Ben‐Arie, Ilan Shelef, Britta Wandschneider, Matthias Koepp, Alon Friedman, Felix Benninger

**Affiliations:** ^1^ Department of Physiology, Professor Vladimir Zelman Inter‐Disciplinary Center of Brain Sciences Ben‐Gurion University of the Negev Beer‐Sheva Israel; ^2^ Department of Cell Biology, Cognitive and Brain Sciences, Professor Vladimir Zelman Inter‐Disciplinary Center of Brain Sciences Ben‐Gurion University of the Negev Beer‐Sheva Israel; ^3^ Department of Neurology Rabin Medical Center, Beilinson Hospital and Tel‐Aviv University Petah Tikva Israel; ^4^ Department of Medical Neuroscience Dalhousie University Halifax Nova Scotia Canada; ^5^ Neurophysiology of Epilepsy Unit National Institute of Neurological Disorders and Stroke, NIH Bethesda Maryland USA; ^6^ Division of Neurosurgery Dalhousie University Halifax Nova Scotia Canada; ^7^ Division of Neurology Dalhousie University Halifax Nova Scotia Canada; ^8^ Academic Unit of Neurology, School of Medicine Trinity College Dublin Ireland; ^9^ FutureNeuro, Science Foundation Ireland Research Centre for Chronic and Rare Neurological Diseases, Royal College of Surgeons in Ireland University of Medicine and Health Sciences Dublin Ireland; ^10^ Smurfit Institute of Genetics Trinity College Dublin Dublin Ireland; ^11^ Department of Diagnostic Radiology Dalhousie University Halifax Nova Scotia Canada; ^12^ Department of Medical Imaging Soroka Medical Center Beer Sheva Israel; ^13^ Department of Clinical and Experimental Epilepsy UCL Queen Square Institute of Neurology London UK; ^14^ UCL‐Epilepsy Society MRI Unit Chalfont Centre for Epilepsy Chalfont St Peter UK

**Keywords:** biomarkers, blood–brain barrier, drug resistance, epilepsy, magnetic resonance imaging

## Abstract

**Objective:**

Blood–brain barrier dysfunction (BBBD) has been linked to various neurological disorders, including epilepsy. This study aims to utilize dynamic contrast‐enhanced magnetic resonance imaging (DCE‐MRI) to identify and compare brain regions with BBBD in patients with epilepsy (PWE) and healthy individuals.

**Methods:**

We scanned 50 drug‐resistant epilepsy (DRE) patients and 58 control participants from four global specialized epilepsy centers using DCE‐MRI. The presence and extent of BBBD were analyzed and compared between PWE and healthy controls.

**Results:**

Both greater brain volume and higher number of brain regions with BBBD were significantly present in PWE compared to healthy controls (*p* < 10^−7^). No differences in total brain volume with BBBD were observed in patients diagnosed with either focal seizures or generalized epilepsy, despite variations in the affected regions. Overall brain volume with BBBD did not differ in PWE with MRI‐visible lesions compared with non‐lesional cases. BBBD was observed in brain regions suspected to be related to the onset of seizures in 82% of patients (*n* = 39) and was typically identified in, adjacent to, and/or in the same hemisphere as the suspected epileptogenic lesion (*n* = 10).

**Significance:**

These findings are consistent with pre‐clinical studies that highlight the role of BBBD in the development of DRE and identify microvascular stabilization as a potential therapeutic strategy.

**Key points**

- Blood–brain barrier dysfunction (BBBD) was observed in more regions and greater total brain volume in epilepsy patients compared to controls.
- BBBD was most often observed in brain regions suspected to be related to the onset of seizures.
- No significant difference in BBBD‐related total brain volume among focal seizures, generalized epilepsy, or magnetic resonance imaging (MRI)–visible lesions in patients with epilepsy (PWE).
- Clobazam was significantly more prevalent in the low‐BBBD group compared to the high‐BBBD group.

## 1 INTRODUCTION

Blood–brain barrier dysfunction (BBBD) has been observed in patients with various neurological disorders, including epilepsy.^1^ The impact of BBBD on neural activity is not fully understood, but studies in rodents have shown that disrupting the BBB or exposure of brain cells to serum proteins (e.g., albumin) leads to activation of the pro‐inflammatory transforming growth factor β (TGFβ) signaling pathway in astrocytes.^2^ This activation leads to changes in astrocytes that compromise their ability to regulate the extracellular environment, including the maintenance of physiological concentrations of potassium and glutamate.^3^, ^4^ In addition, transformed astrocytes release inflammatory cytokines and mediate alterations in the extracellular matrix^5^ and excitatory synaptogenesis,^6^ thereby promoting pathological plasticity^7^ and delayed neurodegeneration.^8^ These cascading effects ultimately lead to dysfunction of the local neural network and the occurrence of spontaneous seizures,^9^ and they are suggested to underlie antiepileptic drug resistance.^7^

Animal studies on the role of BBBD in epileptogenesis and drug‐resistant epilepsy (DRE) have been supported by neuropathological studies using resected tissue from patients with DRE, providing evidence for the presence of iron‐related neuropathology and antioxidant processes in brain tissues, albumin, astrocytic TGF signaling, and neuroinflammation.^10^, ^11^, ^12^, ^13^, ^14^ In addition, seizure‐induced BBBD has been shown in patients with epilepsy (PWE) when scanned up to 180 min following a seizure.^15^ In recent years, dynamic contrast‐enhanced magnetic resonance imaging (DCE‐MRI) has become the standard approach for quantifying the extent and localization of BBBD in humans.^16^ We have recently established and implemented the Veksler linear model, which allows for the detection of a *slow* and more subtle BBB leakage (measured ~6–20 min after contrast injection).^17^, ^18^, ^19^, ^20^ Pre‐clinical and clinical studies confirmed that slow BBBD is quantifiable in various brain pathologies, suggesting a mechanistic association with an increase in trans‐cellular leakage through the compromised barrier.^21^, ^22^, ^23^, ^24^, ^25^, ^26^ In epilepsy, the role of *fast* paracellular leakage (via tight junctions) has also been suggested to underlie BBBD due to downregulation of claudin‐5.^19^ Studies in canines with idiopathic epilepsy further showed prominent BBBD, especially in temporal brain regions, compared with healthy dogs.^27^ A recent study in PWE used comparison between post– and pre–contrast‐enhanced MRI to quantify BBBD, suggesting it is associated with the presumed seizure‐onset zone.^28^ Our objective was to rigorously assess the utility and efficacy of DCE‐MRI in identifying brain regions with BBBD in PWE. This was achieved by implementing a uniform study protocol and centralized blinded analysis across four independent epilepsy centers.

## 2 MATERIALS AND METHODS

### 2.1 Human subjects

The study was approved by the Ethics Committee of Soroka Medical Center, Beer Sheva, Israel (SUMC); St James' Hospital, Dublin, Ireland (SJH); University College London Institute of Neurology and University College London Hospitals, UK (UCLH); and Nova Scotia Health Authority and Dalhousie University, Halifax, Canada (DAL). Written informed consent was obtained from all participants. The study was performed in accordance with the Helsinki Declaration and did not include patients younger than 18 years of age.

### 2.2 Healthy participants

We recruited 58 healthy volunteers (mean age ± standard deviation [SD] = 28.9 ± 5.0, range 19–40; 48.2% male). Forty‐three of them were scanned at SUMC, eight were scanned at DAL, and seven were scanned at UCLH. Inclusion criteria were: (1) age of 18–90 years; and (2) no known history of a neurological, neurosurgical, or psychiatric disorder, and normal kidney functions. We excluded individuals with contraindications for an MRI scan.

### 2.3 Patients with epilepsy

Of 50 PWE (mean age ± SD = 33.6 ± 13.5, range 19–63; 58.0% male) who were recruited to the study, 27 were scanned in SUMC, 11 at DAL, 6 at the SJH, and at 6 at UCLH. Patients were recruited randomly as part of follow‐up in each respective epilepsy clinic. Inclusion criteria were: (1) age of 18–90 years; (2) clinically diagnosed epilepsy according to the International League of Epilepsy (ILAE) 2017 classification^29^; (3) routine laboratory and clinical follow‐ups; (4) at least one electroencephalography (EEG) study interpreted by an epileptologist; (5) classified as DRE, as they continued to have seizures despite appropriate pharmacological treatment with two or more anti‐seizure medications (ASMs; Table 2).

### 2.4 Magnetic resonance imaging

All participants underwent MRI scans utilizing a 3T MRI machine. Scanning was done at least 72 h after the last reported seizure. Initially, unenhanced sequences were acquired (SUMC: T1, fluid‐attenuated inversion recovery [FLAIR] LongTR, T2‐Weighted [T2W] FLAIR, and Driven Equilibrium Single Pulse Observation of T1 [DESPOT1]. SJH: T1, T2, and FLAIR. DAL: T1 and T1W. UCLH: 3Plane Localizer Single Shot Fast Spin Echo [SSFSE], Coronal Magnetization Prepared Rapid Gradient Echo [MPRAGE], Sagittal Cube FLAIR, B1 Field Mapping, and Fast Spoiled Gradient Recalled Echo [FSPGR]. Afterwards, participants underwent a similar DCE‐MRI protocol as previously reported (see Table 1 for more details).^17^

**TABLE 1:** DCE‐MRI acquisition protocols for each institution and the number of scans made.

	SUMC	DAL	UCLH	SJH
3T MRI machine	Philips ingenia	General electric	General electric	Philips achieva
Δt, s	18	20	7.2	22.2
Acquisition duration, min	20	20	20	22
Voxel size, mm	.9 × .9 × 6	1.25 × 1.25 × 6	1.0 × 1.3 ×2.0	.86 ×.86 ×6
Acq. matrix, pixel	192 × 187 ×27	192 × 192 × 34	160 ×160 × 25	256 × 256 × 22
FOV^1^, cm	20	24	25.6	22
DCE: TE/TR,^2^ msec, flip angle (FA), °	FFE,^3^ 2/4, 60	LAVA^4^, 2/4, 15	FSPGR^5^, 5/6.17, 12	FFE, 2.78/5.73, 60
Variable FA, °, TE/TR, msec	5–15–20‐25, 2/10	5–10‐30, 2/10	2–6‐12, 2/6.15	10–15–20‐25‐30, 2.78/5.67
Contrast agent, relaxivity rate, 1/mM·s	Dotarem, 3.89	MultiHance, 6.3	Prohance, 3.09	Dotarem, 3.89
No. of PWE	27	11	6	6
No. of controls	43	8	7	0

Abbreviations: DAL, Nova Scotia Health Authority and Dalhousie University, Halifax, Canada; DCE, dynamic contrast‐enhanced; FFE, fast field echo; FOV, field of view; FSPGR, fast spoiled gradient echo; LAVA, liver acquisition with volume acceleration; PWE, patients with epilepsy; SJH, St James’ Hospital, Dublin, Ireland; SUMC, Soroka Medical Center, Beer Sheva, Israel; TE, echo time; TR, repetition time; UCLH, University College London Institute of Neurology and University College London Hospitals, UK.

^1^ Field of View.

^2^ Echo Time/ Repetition Time.

^3^ Fast Field Echo.

^4^ Liver Acquisition with Volume Acceleration.

^5^ Fast Spoiled Gradient Recalled Echo.

### 2.5 BBB permeability analysis

Images were registered and normalized to Montreal Neurological Institute (MNI) coordinates using Statistical Parametric Mapping (SPM) 12.^30^ Assessment of BBBD was done using in‐house MATLAB scripts, as reported previously.^16^, ^17^, ^21^, ^23^ Briefly, BBB permeability maps were created for all subjects by fitting a linear model and calculating the slope of contrast agent concentration in each voxel from 6 min after injection of the gadolinium‐based contrast agent until the last volume in the DCE‐MRI (Table 1). The analysis script was modified slightly to account for differences in inter‐facility scan protocol in order to ensure an eligible comparison between the facilities (e.g., flip angles, contrast agent relaxivity rate, acquisition duration etc.; Table 1). In addition, to account for inter‐individual variability in intracranial contrast flow, for each region, an individual slope was normalized to the slope measured in the superior sagittal sinus. BBBD was defined as slope values higher than the 95th percentile of all slopes calculated in healthy controls. The percentage of brain volume with supra‐threshold voxels was used as a measure of overall brain volume with BBBD. Region‐specific BBBD analysis was conducted using brain segmentation into 124 regions based on the MNI brain atlas.^31^ The percentage of voxels with supra‐threshold normalized slope was measured for each region. Regions with a median absolute deviation >2 (MAD; modified z‐score) compared to controls were marked as regions with BBBD.

### 2.6 Statistical analysis

Continuous variables are expressed as median ± MAD. Mann–Whitney and Kruskal–Wallis tests were applied for comparison of unpaired nonparametric data sets, accounting for non‐normal distributions. Wilcoxon signed‐rank test was used for paired nonparametric data sets. Categorical variables are reported as frequencies and percentages and were tested by *χ* ^2^ test or Fisher's exact test. Statistical significance was determined when two‐sided *p* ≤ .05. Linear regressions were used to calculate the effect of age and gender on permeability. A one‐way analysis of variance (ANOVA) was used to assess for interactions between age, gender, and permeability. Corrections for multiple comparisons were performed using the Benjamini and Hochberg False Discovery Rate (FDR) method. Effect sizes were calculated using Cohen's *d*. Disease severity was estimated using principal component analysis (PCA) for seizure frequency, number of medications, and years with epilepsy, and was correlated with BBBD using Pearson correlation coefficient.^32^ To test whether BBBD is affected by a specific variable, patients were grouped into high and low BBBD based on % volume with BBBD above/below median. This grouping method, based on the median value, ensures a balanced comparison between the groups, providing a more representative analysis of the ASM's effect on BBBD in the patient population.^33^

## 3 RESULTS

### 3.1 BBBD is more prevalent in PWE

No significant differences were observed in age (*p* = .39) or gender (*p* = .29) between PWE and controls. Of 50 PWE, 40 had a clinical diagnosis of focal‐onset seizures (mean age ± SD 34.3 ± 13.2 years, 65% male; Table 2), and 10 were clinically diagnosed with generalized seizures (mean age ± SD 30.8 ± 14.8 years, 30% male). Based on semiology and/or long‐term EEG monitoring findings, the seizure‐onset zone was suspected to be in the temporal lobe for 24 PWE (mean age ± SD 32.5 ± 12.3 years, 66.7% male), in the frontal lobe for 14 PWE (mean age ± SD 38.2 ± 15.7 years, 50% male), one in the parietal lobe and one undetermined.

**TABLE 2:** Demographics and clinical data.

	Controls *N =* 58	Epilepsy *N =* 50	Focal *n =* 40	Generalized *n =* 10	Temporal *n =* 24
Gender, female %	51.72	40	32.5	70	31.82
Age, years (SD)	28.94 (5.08)	33.64 (13.51)	34.35 (13.25)	30.80 (14.87)	31.95 (12.70)
Age at onset, median, years (range)		16.50 (1.5–63)	17.00 (1.5–63)	13.00 (3–23)	17.00 (2–31)
Epilepsy duration, median, years (range)		15.00 (0–55)	14.50 (0–44)	17.00 (0–55)	13.00 (0–44)
Polytherapy %		66	75	30	81.81
Lesional %		46.29	45	30	59.09

**FIGURE 1:**
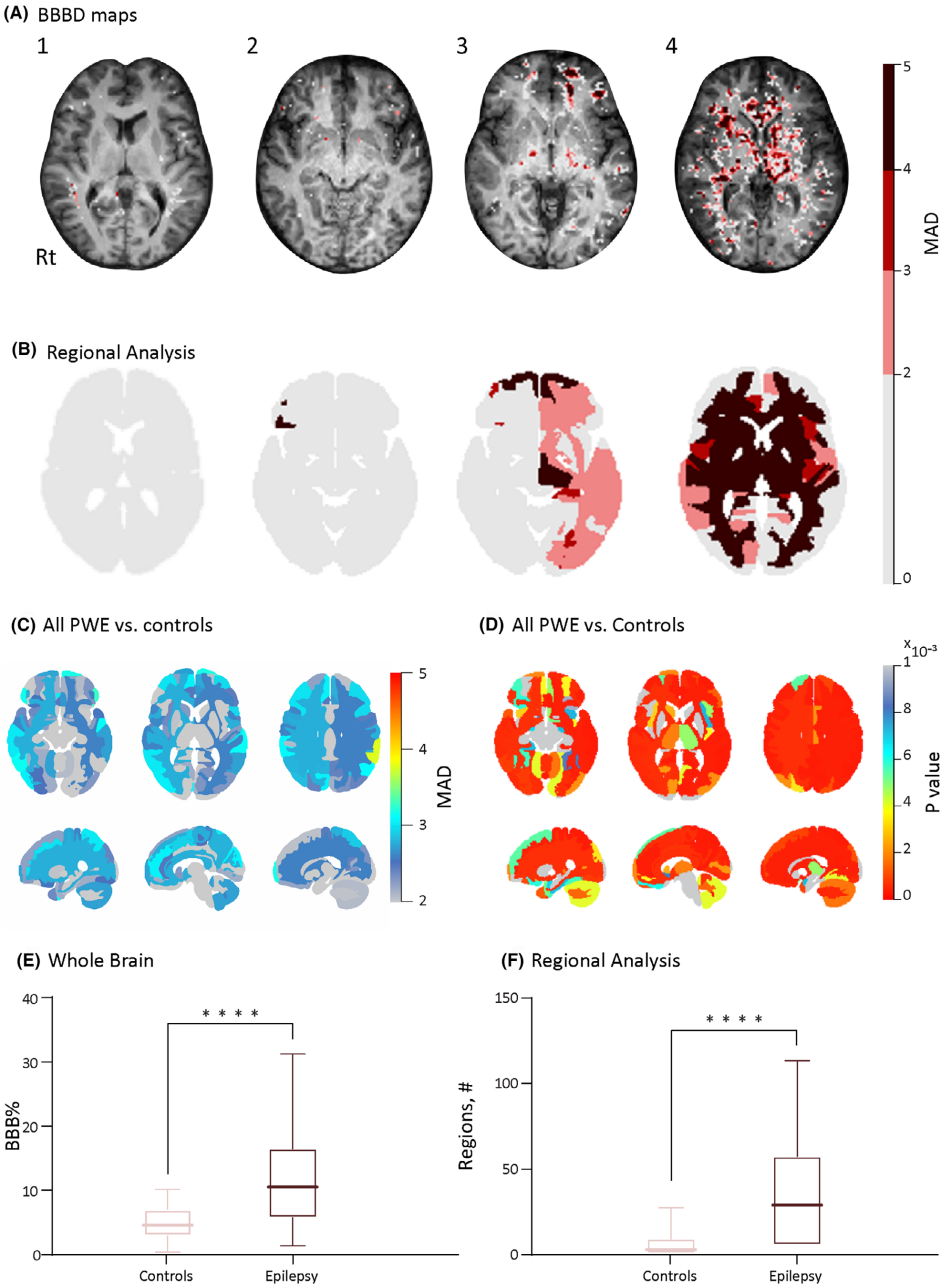
Epilepsy vs controls. DCE‐MRI reveals persistent BBBD in patients with epilepsy. (A) Examples of patients’ BBBD analysis results. 1, Healthy control. 2, Left temporal lobe epilepsy. 3, Left frontal lobe epilepsy. 4, Generalized epilepsy. (B) Examples of MAD 124 regions BBBD maps. The MAD between 0 and 2 is transparent. (C) Average MAD above controls in regional maps of 124 areas. Left–right lobe comparison was found to be insignificant (*p* = .13). (D) Significant regions of patients with epilepsy compared to controls with FDR applied (*p* < .001). (E) Statistics of BBB% between controls and patients with epilepsy (*p* < 10^−7^). (F) Statistics of percentage of regions with BBBD between controls and patients with epilepsy (*p* < 10^−8^). Error bars indicate standard deviation (SD). BBBD, blood brain barrier dysfunction; DCE‐MRI, dynamic contrast enhanced magnetic resonance imaging; FDR, false discovery rate; MAD, mean absolute deviation; SD, standard deviation.

Permeability maps generated for each subject were superimposed on T1 images to reveal BBBD as voxels with a MAD >2 compared with control data (Figure 1A). PWE had a higher average percentage of brain voxels with BBBD compared with controls (11.7 ± 7.2% vs 4.8 ± 2.6%, *p* < 10^−7^; Figure 1E) and higher average number of regions with BBBD (57.1 ± 35.2 vs 15.3 ± 14, *p* < 10^−8^; Figure 1F).

### 3.2 Regional analysis reveals brain regions more susceptible to BBBD

Regional analysis for BBBD localization identified distinctly permeable brain regions after applying FDR correction (see Section 2 and Figure 1A–D). In 6 of 50 PWE (12%) and in 15 of 58 controls (25.9%, *p* = .4), no brain regions with BBBD were identified. We noted substantial variability among PWE in terms of the degree and location of BBBD. A per‐group comparison showed an average MAD >2 in 15 brain regions (mostly cortical gray matter), of which 9 were within the frontal and temporal lobes (Figure 1C). These regions included the left supramarginal gyrus (MAD = 4.02), left orbital part of the inferior frontal gyrus (3.6), left postcentral gyrus medial segment (3.54), left superior temporal gyrus (3.48), right orbital part of the inferior frontal gyrus (3.46), left frontal pole (3.4), left opercular part of the inferior frontal gyrus (3.35), right superior parietal lobule (3.29), and the right accumbens area (3.28). Except for the right occipital pole, an FDR‐corrected group comparison showed significantly higher permeability in 123 of 124 brain regions in PWE compared with controls (Figure 1D).

**FIGURE 2:**
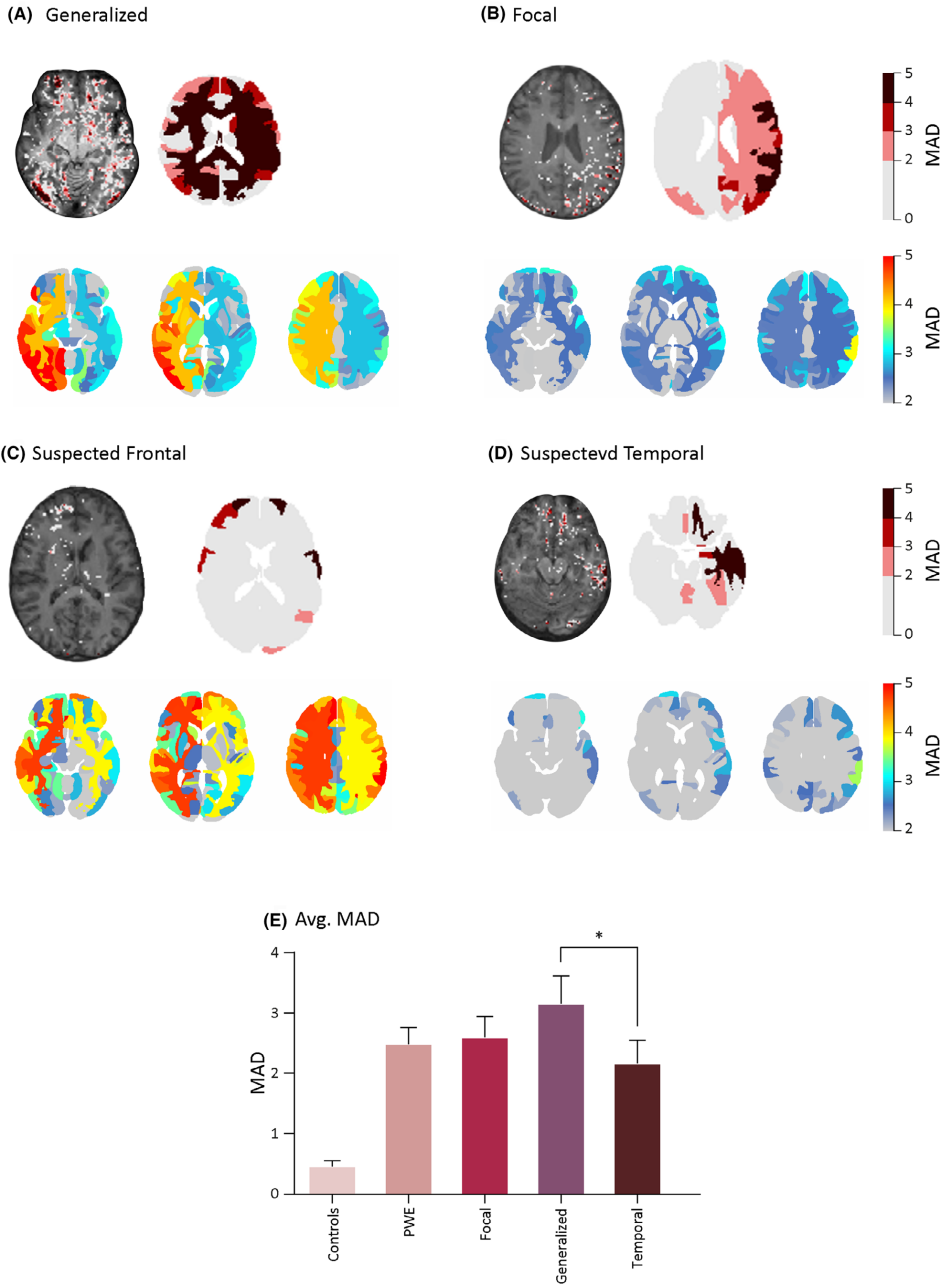
Regional analysis. Imaging patients with epilepsy reveals blood–brain barrier dysfunction. (A–D). Upper images: Example of a T1 image (left image) and a regional analysis (right) in a representative patient with: (A) generalized epilepsy (left–right lobe comparison, *p* < 10^−7^), (B) focal epilepsy (*p* = .68), (C) suspected frontal epilepsy (*p* = .02), and (D) suspected temporal epilepsy (*p* = .13). Lower images: Average MAD maps per region in PWE, categorized into the same groups as the upper images. (E) Average MAD scores across all patients in each group for all regions (*p = .04). MAD, mean absolute deviation; PWE, patients with epilepsy.

No significant differences were found in the average number of brain regions with BBBD between patients with focal vs generalized epilepsy (59.0 ± 35.9 vs 71.3 ± 29.0, respectively, *p* = .2; Figure 2E). Notably, compared to controls, regional analysis revealed 19 regions with BBBD in patients with focal epilepsy, whereas the generalized epilepsy group 43 regions showed BBBD (Figure 2A,B; Table S2). Left–right hemispheres average MAD asymmetry comparison found significant differences in generalized PWE (2.6 ± .7 vs 3.7 ± 1.1, *p* < 10^−7^, *d* = 1.1; Figure 2A) and in frontal PWE (3.2 ± 1.1 vs 3.6 ± .9, *p* = .02, *d* = .43; Figure 2C).

Suspected frontal and temporal PWE average number of regions with BBBD comparison did not show significant differences (70.2 ± 41.8 vs 54.6 ± 30.3 respectively, *p* = .1). Noteworthy, regions showing the highest BBBD are the left supramarginal gyrus (frontal MAD = 5.7, temporal MAD = 4.4) and the left postcentral gyrus medial segment (5.4, 4.2; Figure 2C,D). In addition, a higher degree of BBBD (>2 MAD of controls) group comparison found a significant difference between generalized and temporal PWE (3.1 ± 1.4 vs 2.1 ± 1.6, *p* = .04; Figure 2E).

### 3.3 BBBD in patients with MR‐positive lesions

**FIGURE 3:**
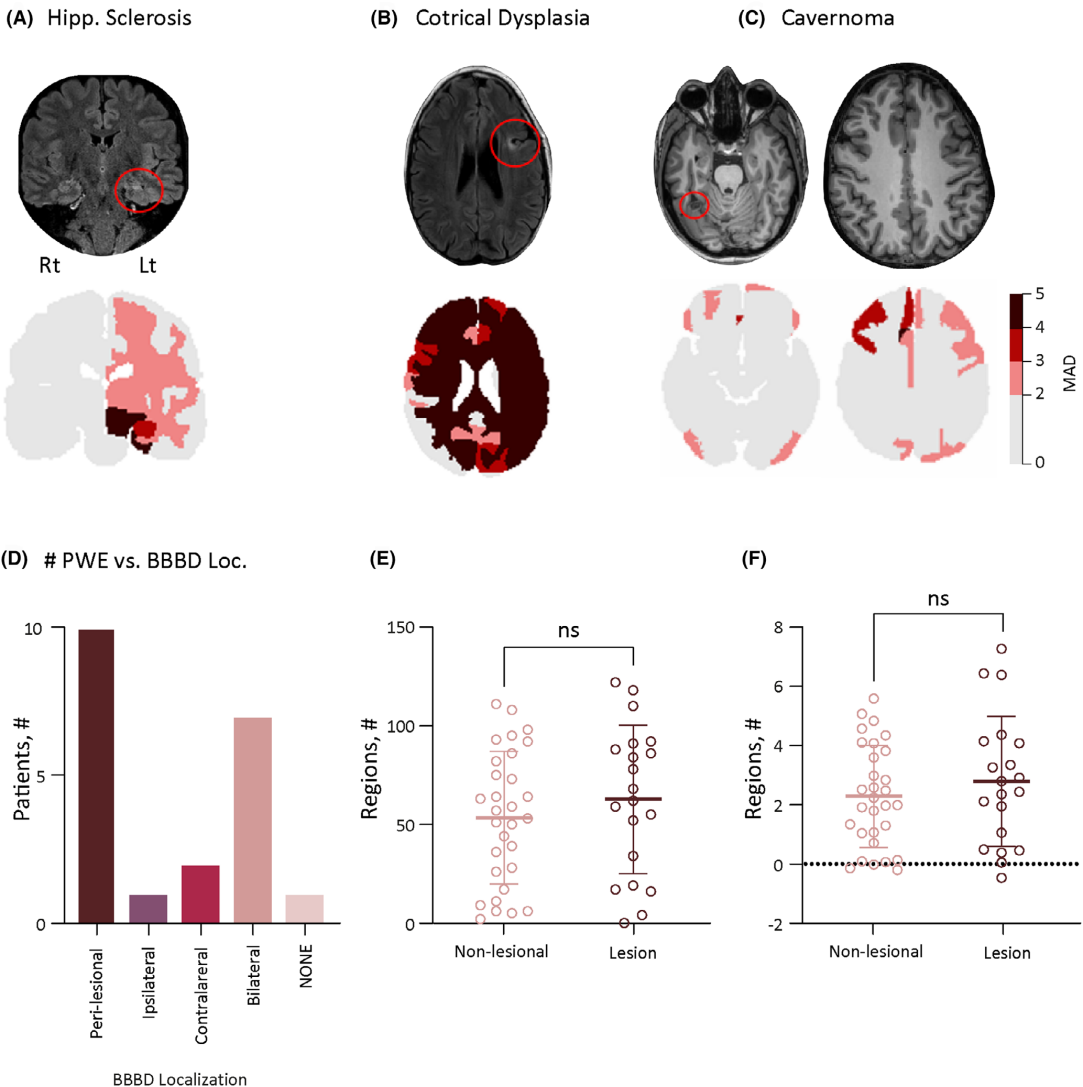
Lesion and BBBD. Lesions in MR are associated with blood–brain barrier dysfunction (BBBD). BBBD in patients with a “lesional MRI”: (A) A 35‐year‐old man with left hippocampal sclerosis. (B) A 33‐year‐old man with left frontal cortical dysplasia. (C) A 20‐year‐old woman with right occipital cavernoma. Upper row: Representative anatomic images showing the lesion (red circle). Lower row: Regional analysis of BBB leakage. (D) Location of region(s) with highest BBBD in relation to lesion site (*p* = .49) (see Table S1). (E) Number of brain regions with BBBD (MAD >2) in patients with and without MRI‐positive lesion. (F) Average MAD in patients with and without MRI‐positive lesion (*p* = .41). BBBD, blood brain barrier dysfunction; MAD, mean absolute deviation; MR, magnetic resonance.

In 21 PWE, neuroradiological assessment of structural MRI revealed a visible pathology, including temporal (*n* = 11), frontal (*n* = 4), parietal (*n* = 4), and occipital (*n* = 2). BBBD was most often found ipsilateral and adjacent to the lesion (*n* = 10, Figure 3A). However, in seven patients, BBBD was found in both the ipsilateral and contralateral hemispheres (Figure 3B). In one patient, BBBD was ipsilateral to the lesion (seemingly unrelated, Figure 3C), or in the contralateral hemisphere (Figure 3D, *n* = 2). In one patient with mesial temporal sclerosis, no BBBD could be detected (Figure 3D). No significant difference was found in the number of regions with BBBD between patients with lesions and those without visible pathology (62.7 ± 37.6 vs 53.4 ± 33.6, *p* = .4), and no significant difference was observed in the average MAD (2.7 ± 2.2 vs 2.2 ± 1.7, *p* = .4; Figure 3E,F).

### 3.4 Medications

All patients in this cohort were treated with ASMs (Table S3). No significant differences were found in the extent (brain volume with BBBD or averaged modified *Z* score) or the number of regions with BBBD in patients who were prescribed one (*n* = 21), two (*n* = 14), or more (*n* = 19) different ASMs (42.6 ± 28.6, 22.9 ± 25.3, and 41.4 ± 34.6, respectively, *p* = .08; modified *Z* score 1.6 ± 1.3, .7 ± 1.0, and 1.5 ± 1.3, *p* = .1). To test whether the BBBD is affected by a specific ASM, patients were grouped into high and low BBBD (10.4% threshold; high BBBD: *n* = 25, 17.5 ± 5.4%; low BBBD: *n* = 25, 6.0 ± 3.1%). No differences were found between the groups in the number of prescribed ASMs or any single drug, except in patients treated with clobazam—who were more frequent in the low‐BBBD group (*p* = .01 Figure S2, *n* = 10).

Furthermore, we did not identify a significant correlation between BBBD and tonic‐clonic seizures (*n* = 28) compared to other types of seizures (e.g., absence, focal seizures, and unknown; *n* = 22; *p* = .8), age (*p* = .8), age at epilepsy onset (*p* = .2), year with epilepsy (*p* = .1), or seizure frequency (*p* = .3). The correlation with gender was nearly significant (*p* = .07; Figure S1). We further used PCA to estimate epilepsy severity as reported (see Methods).^32^ A moderate positive Spearman correlation of .22 (*p* = .1) was found between PC1 (explained variance 63.9%) and the number of regions with BBBD, and a slight negative correlation of −.19 with PC2 (explain variance 35.4%; *p* = .1) and the number of regions with BBBD, with no significant correlation with % brain volume with BBBD.

## 4 DISCUSSION

In this multicenter prospective study, we present evidence of interictal BBBD in patients with DRE. Our findings extend the insights gained from prior imaging studies in canine models of epilepsy and are consistent with findings in rodent models.^27^ Although BBBD has been shown previously in DCE‐MRI studies in various neurological conditions, its impact on disease presentation and progression remains unknown. We demonstrated previously that patients with transient ischemic attack or minor stroke who have a compromised BBB are at a greater risk of developing recurrent stroke^21^ and post‐traumatic epilepsy.^8^ Patients with bipolar disorder and extensive BBBD were more likely to exhibit disease progression,^22^ and patients with systemic lupus erythematosus and BBBD were more likely to demonstrate cognitive deficits.^23^

Our method was developed to delineate the extent and spatial distribution of BBBD in individual PWE, with the goal of proposing a new diagnostic and prognostic biomarker in epilepsy. Although statistical comparison between PWE and controls show increased distribution of BBBD extent in 123 of 124 regions, a higher degree of BBBD (>2 MAD of controls) was seen particularly in frontal and temporal cortical gray matter (Table S2). This aligns with findings in rodent models of temporal lobe epilepsy and spontaneous canine epilepsy,^26^, ^27^ potentially supporting a link between BBBD and epileptogenesis, particularly in regions with a lower seizure threshold. Evidence of BBBD adjacent to MR‐detected epileptogenic lesions (e.g., hippocampal sclerosis; Figure 3) further supports this association.

For some patients, BBBD could be detected independently in multiple brain regions, an entire hemisphere, or both hemispheres (Figure 1A). Although these findings do not exclude an epileptogenic process in regions outside the clinically suspected seizure‐onset zone, a compromised BBB in brain regions outside the seizures‐onset zone may highlight potential propagation pathways and/or dual intracranial pathology and/or mechanism underlying comorbidities. In addition, we cannot rule out that a more widespread BBBD was due to recent subclinical or unreported seizures,^15^ metabolic aberrancies,^22^ or a small vessel disease.^21^ Studies including larger cohorts should explore the impact of BBBD on epilepsy comorbidities, such as mood‐related disorders, and the coexistence of metabolic or hormonal disorders. Future studies on the association between BBBD extent and spatial distribution, intracranial EEG recordings, and postoperative seizure freedom are expected to shed light on the role of DCE‐MRI in the pre‐surgical assessment.

Of interest, we did not find differences in the extent of BBBD between patients diagnosed with focal or generalized epilepsy, which may be attributed to different etiologies (e.g., inflammatory, genetic, or structural) and the proximity to a seizure. Notably, higher BBBD (average MAD) was found in patients with generalized epilepsy compared to those with temporal epilepsy. Despite finding no difference in the number of regions with BBBD between these groups, the higher MAD suggests a greater degree of leakage. This increased leakage may contribute to a faster propagation of epileptic discharges in patients with generalized epilepsy. In addition, the left–right hemispheres comparison showed a significant difference in sides for generalized epilepsy and suspected frontal epilepsy, which might be attributed to the low sample sizes (*n* = 10 and *n* = 14, respectively; Figure 2A,B). These findings warrant further investigation in future studies with larger sample sizes.

The high prevalence of BBBD in our cohort could be influenced by a sampling bias of patients who experienced frequent seizures despite treatment with multiple ASMs. If such confounding exists in our cohort, the high BBBD occurrence could be related to high seizure frequency, effect of drugs, or related to DRE. However, in our analysis, no significant correlation was found between BBBD extent (brain volume) and seizure frequency. Future studies should investigate BBBD in patients who are seizure‐free or prior to starting ASMs. In addition, we found no correlation between the number of regions with BBBD or spatial distribution and seizure frequency or the number of prescribed ASMs.

Of interest, a small group of patients (*n* = 10) who were treated with the benzodiazepine, clobazam, were predominantly present in the low BBBD group (<50% of brain volume with BBBD, *n* = 8). Whether benzodiazepines have a direct effect on BBB integrity is not known^34^, ^35^ and may be a subject for future studies.

BBBD in experimental animals and surgically resected PWE tissue has been shown previously to be associated with the presence of serum albumin within the brain neuropil. Of interest, exposing brain tissue to serum albumin in rodents was shown to acutely reduce seizure threshold,^36^ induce epileptogenesis,^6^, ^26^, ^37^ and underlie resistance to anti‐seizure medications in brain slices exposed to 4‐aminopyridine (4‐AP).^38^ Although the mechanisms of BBBD in drug resistance are not known, they could be associated with reduced levels of free ASMs due to binding to albumin and/or other vascular pathologies underlying enhanced drug transport.^39^ Our study does support, however, the notion that therapeutics targeting BBBD may become a novel approach for treating DRE.

Our findings confirm the utility of DCE‐MRI in assessing BBBD, aligning with the results reported previously.^28^ In addition to corroborating their observations, our study provides a more nuanced analysis of individual variability, regional differences, and comparative data with healthy controls.^17^ This detailed approach enhances the understanding of the underlying mechanisms driving BBBD, offering a broader perspective on how permeability alterations manifest in PWE. The findings that age, gender, age of onset of epilepsy, years with epilepsy, and seizure frequency measurements were insignificant suggest that these factors do not significantly impact the BBBD. Although an effort was made to avoid imaging in the first 48 h after seizure, we cannot rule out a transient effect of seizures on the BBBD in our cohort.^15^ Disparities were found between certain facilities, whereas no notable differences were observed in control site comparisons. However, the small sample size and patient variability warrant caution when interpreting these results, especially between subgroups. In addition, BBBD was classified as permeable or non‐permeable, which may oversimplify a complex condition, suggesting the need for a detailed analysis on the extent and nature of permeability changes in future studies as well as larger cohorts and patients who undergo resection surgery.

In conclusion, our quantitative assessment of the extent and spatial distribution of BBBD in PWE has confirmed a high prevalence of compromised BBB integrity. These findings endorse DCE‐MRI as an additional tool for identifying and potentially localizing the seizure‐onset zone in pre‐surgical assessments. Larger studies are necessary to uncover the role of BBB imaging in diagnosing, prognosticating, evaluating pharmacological responsiveness, and determining surgical outcomes in PWE.

## FUNDING INFORMATION

This work was supported by European Research Area Network NeuronAward (Canadian Institutes of Health Research (CIHR) Award no. NDD 168164) (Alon Friedman [A.F.]); Neurosurgery Research Education Foundation and Academy of Neurological Surgeons Research Fellowship Grant (Mark A. Maclean [M.A.M.] and A.F.); CIHR Project Grant (grant number PJT‐180636, 488048) (M.A.M., Britta Wandschneider [B.Wh.], and A.F.); Israel Science Foundation (grant no.. 2254/20) (A.F.); and Binational Israel‐USA Science Foundation grant (A.F and Fexlix Benninger [F.B]). This work was supported in part by a Science Foundation Ireland (SFI) (Eye‐D‐21/SPP/3732), The Irish Research Council (IRC), and by a research grant from SFI under grant number 16/RC/3948, and co‐funded under the European Regional Development fund by FutureNeuro industry partners. The Campbell lab is also supported by a European Research Council (ERC) grant, “Retina‐Rhythm” (864522). UCLH funding was supported by the Medical Research Council (MRC) (MR/T005335/1).

## CONFLICT OF INTEREST STATEMENT

None of the authors has any conflict of interest to disclose.

## ETHICS STATEMENT

We confirm that we have read the Journal's position on issues involved in ethical publication and affirm that this report is consistent with those guidelines.

## Supporting information


Data S1.

## Data Availability

The data that support the findings of this study are available from the corresponding author upon reasonable request.

## References

[1] Serlin Y , Shelef I , Knyazer B , Friedman A . Anatomy and physiology of the blood‐brain barrier. Semin Cell Dev Biol. 2015;38:2–6. 25681530 10.1016/j.semcdb.2015.01.002 PMC4397166

[2] Heinemann U , Kaufer D , Friedman A . Blood‐brain barrier dysfunction, TGFβ signaling, and astrocyte dysfunction in epilepsy. Glia. 2012;60(8):1251–1257. 22378298 10.1002/glia.22311 PMC3615248

[3] Cacheaux LP , Ivens S , David Y , Lakhter AJ , Bar‐Klein G , Shapira M , et al. Transcriptome profiling reveals TGF‐beta signaling involvement in epileptogenesis. J Neurosci. 2009;29(28):8927–8935. 19605630 10.1523/JNEUROSCI.0430-09.2009 PMC2875073

[4] David Y , Cacheaux LP , Ivens S , Lapilover E , Heinemann U , Kaufer D , et al. Astrocytic dysfunction in epileptogenesis: consequence of altered potassium and glutamate homeostasis? J Neurosci. 2009;29(34):10588–10599. 19710312 10.1523/JNEUROSCI.2323-09.2009 PMC2875068

[5] Kim SY , Porter BE , Friedman A , Kaufer D . A potential role for glia‐derived extracellular matrix remodeling in postinjury epilepsy. J Neurosci Res. 2016;94(9):794–803. 27265805 10.1002/jnr.23758

[6] Weissberg I , Wood L , Kamintsky L , Vazquez O , Milikovsky DZ , Alexander A , et al. Albumin induces excitatory synaptogenesis through astrocytic TGF‐β/ALK5 signaling in a model of acquired epilepsy following blood–brain barrier dysfunction. Neurobiol Dis. 2015;78:115–125. 10.1016/j.nbd.2015.02.029 25836421 PMC4426044

[7] Salar S , Lapilover E , Müller J , Hollnagel JO , Lippmann K , Friedman A , et al. Synaptic plasticity in area CA1 of rat hippocampal slices following intraventricular application of albumin. Neurobiol Dis. 2016;91:155–165. 26972679 10.1016/j.nbd.2016.03.008

[8] Tomkins O , Friedman O , Ivens S , Reiffurth C , Major S , Dreier JP , et al. Blood‐brain barrier disruption results in delayed functional and structural alterations in the rat neocortex. Neurobiol Dis. 2007;25(2):367–377. 17188501 10.1016/j.nbd.2006.10.006

[9] Bar‐Klein G , Cacheaux LP , Kamintsky L , Prager O , Weissberg I , Schoknecht K , et al. Losartan prevents acquired epilepsy via TGF‐β signaling suppression. Ann Neurol. 2014;75(6):864–875. 24659129 10.1002/ana.24147 PMC4077937

[10] van Vliet EA , Aronica E , Gorter JA . Role of blood‐brain barrier in temporal lobe epilepsy and pharmacoresistance. Neuroscience. 2014;277:455–473. 25080160 10.1016/j.neuroscience.2014.07.030

[11] Kim SY , Senatorov VV Jr , Morrissey CS , Lippmann K , Vazquez O , Milikovsky DZ , et al. TGFβ signaling is associated with changes in inflammatory gene expression and perineuronal net degradation around inhibitory neurons following various neurological insults. Sci Rep. 2017;7(1):7711. 28794441 10.1038/s41598-017-07394-3 PMC5550510

[12] Schmitz AK , Grote A , Raabe A , Urbach H , Friedman A , von Lehe M , et al. Albumin storage in neoplastic astroglial elements of gangliogliomas. Seizure. 2013;22(2):144–150. 23182422 10.1016/j.seizure.2012.10.014 PMC3646260

[13] Raabe A , Schmitz AK , Pernhorst K , Grote A , von der Brelie C , Urbach H , et al. Cliniconeuropathologic correlations show astroglial albumin storage as a common factor in epileptogenic vascular lesions. Epilepsia. 2012;53(3):539–548. 22372630 10.1111/j.1528-1167.2012.03405.x PMC3669690

[14] Zimmer TS , David B , Broekaart DWM , Schidlowski M , Ruffolo G , Korotkov A , et al. Seizure‐mediated iron accumulation and dysregulated iron metabolism after status epilepticus and in temporal lobe epilepsy. Acta Neuropathol. 2021;142(4):729–759. 34292399 10.1007/s00401-021-02348-6 PMC8423709

[15] Rüber T , David B , Lüchters G , Nass RD , Friedman A , Surges R , et al. Evidence for peri‐ictal blood‐brain barrier dysfunction in patients with epilepsy. Brain. 2018;141(10):2952–2965. 30239618 10.1093/brain/awy242

[16] Chassidim Y , Veksler R , Lublinsky S , Pell GS , Friedman A , Shelef I . Quantitative imaging assessment of blood‐brain barrier permeability in humans. Fluids Barriers CNS. 2013;10(1):9. 23388348 10.1186/2045-8118-10-9 PMC3570379

[17] Veksler R , Vazana U , Serlin Y , Prager O , Ofer J , Shemen N , et al. Slow blood‐to‐brain transport underlies enduring barrier dysfunction in American football players. Brain. 2020;143(6):1826–1842. 10.1093/brain/awaa140 32464655 PMC7297017

[18] O'Keeffe E , Kelly E , Liu Y , Giordano C , Wallace E , Hynes M , et al. Dynamic blood‐brain barrier regulation in mild traumatic brain injury. J Neurotrauma. 2020;37(2):347–356. 31702476 10.1089/neu.2019.6483 PMC10331162

[19] Greene C , Hanley N , Reschke CR , Reddy A , Mäe MA , Connolly R , et al. Microvascular stabilization via blood‐brain barrier regulation prevents seizure activity. Nat Commun. 2022;13(1):2003. 35422069 10.1038/s41467-022-29657-y PMC9010415

[20] Ware JB , Sinha S , Morrison J , Walter AE , Gugger JJ , Schneider ALC , et al. Dynamic contrast enhanced MRI for characterization of blood‐brain‐barrier dysfunction after traumatic brain injury. Neuroimage Clin. 2022;36:103236. 36274377 10.1016/j.nicl.2022.103236 PMC9668646

[21] Serlin Y , Ofer J , Ben‐Arie G , Veksler R , Ifergane G , Shelef I , et al. Blood‐brain barrier leakage: a new biomarker in transient ischemic attacks. Stroke. 2019;50(5):1266–1269. 31009340 10.1161/STROKEAHA.119.025247

[22] Kamintsky L , Cairns KA , Veksler R , Bowen C , Beyea SD , Friedman A , et al. Blood‐brain barrier imaging as a potential biomarker for bipolar disorder progression. Neuroimage Clin. 2020;26:102049. 31718955 10.1016/j.nicl.2019.102049 PMC7229352

[23] Kamintsky L , Beyea SD , Fisk JD , Hashmi JA , Omisade A , Calkin C , et al. Blood‐brain barrier leakage in systemic lupus erythematosus is associated with gray matter loss and cognitive impairment. Ann Rheum Dis. 2020;79(12):1580–1587. 33004325 10.1136/annrheumdis-2020-218004

[24] Weissberg I , Veksler R , Kamintsky L , Saar‐Ashkenazy R , Milikovsky DZ , Shelef I , et al. Imaging blood‐brain barrier dysfunction in football players. JAMA Neurol. 2014;71(11):1453–1455. 25383774 10.1001/jamaneurol.2014.2682

[25] Vazana U , Veksler R , Pell GS , Prager O , Fassler M , Chassidim Y , et al. Glutamate‐mediated blood‐brain barrier opening: implications for neuroprotection and drug delivery. J Neurosci. 2016;36(29):7727–7739. 27445149 10.1523/JNEUROSCI.0587-16.2016 PMC4951577

[26] Bar‐Klein G , Lublinsky S , Kamintsky L , Noyman I , Veksler R , Dalipaj H , et al. Imaging blood‐brain barrier dysfunction as a biomarker for epileptogenesis. Brain. 2017;140(6):1692–1705. 28444141 10.1093/brain/awx073

[27] Hanael E , Veksler R , Friedman A , Bar‐Klein G , Senatorov VV Jr , Kaufer D , et al. Blood‐brain barrier dysfunction in canine epileptic seizures detected by dynamic contrast‐enhanced magnetic resonance imaging. Epilepsia. 2019;60(5):1005–1016. 31032909 10.1111/epi.14739

[28] Reiter JT , Schulte F , Bauer T , et al. Evidence for interictal blood‐brain barrier dysfunction in people with epilepsy. Epilepsia. 2024;4:1462–1474. 10.1111/epi.17929 38436479

[29] Fisher RS , Cross JH , D'Souza C , French JA , Haut SR , Higurashi N , et al. Instruction manual for the ILAE 2017 operational classification of seizure types. Epilepsia. 2017;58(4):531–542. 28276064 10.1111/epi.13671

[30] Statistical Parametric Mapping. Accessed March 10, 2024. https://www.fil.ion.ucl.ac.uk/spm/

[31] spm12/tpm at master · neurodebian/spm12. GitHub. Accessed March 10, 2024. https://github.com/neurodebian/spm12/tree/master/tpm

[32] Xiao F , Caciagli L , Wandschneider B , Sone D , Young AL , Vos SB , et al. Identification of different MRI atrophy progression trajectories in epilepsy by subtype and stage inference. Brain. 2023;146(11):4702–4716. 37807084 10.1093/brain/awad284 PMC10629797

[33] Iacobucci D , Posavac SS , Kardes FR , Schneider MJ , Popovich DL . The median Split: robust, refined, and revived. J Consum Psychol. 2016;25(4), 690–704.

[34] Swissa E , Bar‐Klein G , Serlin Y , Weissberg I , Kamintsky L , Eisenkraft A , et al. Midazolam and isoflurane combination reduces late brain damage in the paraoxon‐induced status epilepticus rat model. Neurotoxicology. 2020;78:99–105. 32084435 10.1016/j.neuro.2020.02.007

[35] Zheng J , Zhang W , Kang P , Zheng X , He K , Bai H , et al. Midazolam ameliorates impairment of the blood‐brain barrier (BBB) against LPS. Neurotox Res. 2022;40(3):751–762. 35451708 10.1007/s12640-022-00508-4

[36] Frigerio F , Frasca A , Weissberg I , Parrella S , Friedman A , Vezzani A , et al. Long‐lasting pro‐ictogenic effects induced in vivo by rat brain exposure to serum albumin in the absence of concomitant pathology. Epilepsia. 2012;53(11):1887–1897. 22984896 10.1111/j.1528-1167.2012.03666.x PMC3651831

[37] Ivens S , Kaufer D , Flores LP , Bechmann I , Zumsteg D , Tomkins O , et al. TGF‐beta receptor‐mediated albumin uptake into astrocytes is involved in neocortical epileptogenesis. Brain. 2007;130(Pt 2):535–547. 17121744 10.1093/brain/awl317

[38] Salar S , Maslarova A , Lippmann K , Nichtweiss J , Weissberg I , Sheintuch L , et al. Blood‐brain barrier dysfunction can contribute to pharmacoresistance of seizures. Epilepsia. 2014;55(8):1255–1263. 24995798 10.1111/epi.12713

[39] Liu JYW , Thom M , Catarino CB , Martinian L , Figarella‐Branger D , Bartolomei F , et al. Neuropathology of the blood‐brain barrier and pharmaco‐resistance in human epilepsy. Brain. 2012;135(Pt 10):3115–3133. 22750659 10.1093/brain/aws147

