# Supplementary material for: Imaging blood–brain barrier dysfunction in drug‐resistant epilepsy: A multi‐center feasibility study
Source: Epilepsia. 2024 Nov 6;66(1):195–206. doi: 10.1111/epi.18145 (PMC11742632; doi:10.1111/epi.18145)
Supplement: Supplementary file 1 — Data S1. [file EPI-66-195-s001.docx]

**Imaging Blood-Brain Barrier Dysfunction in Drug-Resistant Epilepsy: A Multi-Center Feasibility Study**

**Supplementary Materials**

**Supplementary Results**

PWE facility comparison revealed significant differences between SUMC (*n =* 27) and DAL (*n =* 11) (*p* < 0.001), as well as between SUMC and UCLH (*n =* 6) (*p* < 0.001). In contrast, comparisons between SUMC and SJH (*n =* 6) (*p* = 0.21), DAL and SJH (*p* = 0.07), and DAL and UCLH (*p* = 0.87) did not demonstrate significant differences. However, a difference was observed between SJH and UCLH (*p* = 0.04). Control facility comparison revealed no significant differences between SUMC (*n =* 43) and UCLH (*n =* 7) (*p* = 0.67), SUMC and DAL (*n =* 8; *p* = 0.13), or UCLH and DAL (*p* = 0.67).

In examining the correlation between epilepsy severity and its features; seizure frequency per month, number of medications, and year of epilepsy onset, the analysis revealed a Spearman correlation of 0.22 between principal component 1 (PC1) and the number of regions, and a -0.19 correlation between principal component 2 (PC2) and the number of regions. Features; seizure frequency per month, number of medications, age of onset, year of epilepsy diagnosis, age, and presence of lesions, the findings indicated a Spearman correlation of 0.02 between principal component 1 (PC1) and the number of regions, and a more pronounced -0.3 correlation between principal component 2 (PC2) and the number of regions.

SHapley Additive exPlanations (SHAP) analysis was employed to assess the impact of each feature on the model's predictions for the number of regions with BBBD, taking into account the interaction with other features. Comparing features Focal/General, Focal Type, age, gender, Epilepsy type, Seizures type, number of medications, type of medication, family history, age of onset, year of epilepsy, seizure frequency, lesion, EEG to the numbers of regions with BBBD identified the most significant features to predict the value of the number of regions with BBBD is: age: 6.64, age of onset: 3.46, seizure frequency: 5.12, number of medication: 6.03, epilepsy type refractory TLE: 4.44.

A comparison of frontal and temporal regions between ipsilateral and contralateral sides in PWE revealed no significant differences (*p* = 0.7). Left frontal operculum: (ipsilateral = 4.66, contralateral = 0.72); Left frontal pole: (3.30, 2.88); Left inferior temporal gyrus: (2.16, 1.83); Left medial frontal cortex: (4.22, 2.77); Left middle frontal gyrus: (4.76, 5.96); Left superior frontal gyrus medial segment: (4.40, 4.75); Left middle temporal gyrus: (2.98, 1.79); Left opercular part of the inferior frontal gyrus: (6.70, 5.68); Left orbital part of the inferior frontal gyrus: (3.43, 4.18); Left planum temporale: (2.52, 2.61); Left superior frontal gyrus: (5.35, 4.99); Left superior temporal gyrus: (3.40, 2.61); Left temporal pole: (2.70, 2.84); Left triangular part of the inferior frontal gyrus: (6.05, 2.86); Left transverse temporal gyrus: (2.12, 2.60); Right frontal operculum: (0.18, 3.59); Right frontal pole: (4.08, 3.51); Right inferior temporal gyrus: (1.99, 2.24); Right medial frontal cortex: (3.69, 4.49); Right middle frontal gyrus: (5.22, 5.75); Right superior frontal gyrus medial segment: (7.49, 4.92); Right middle temporal gyrus: (1.38, 2.36); Right opercular part of the inferior frontal gyrus: (4.64, 4.38); Right orbital part of the inferior frontal gyrus: (1.20, 4.76); Right planum temporale: (1.54, 2.35); Right superior frontal gyrus: (4.72, 5.13); Right superior temporal gyrus: (1.25, 2.01); Right temporal pole: (1.62, 1.91); Right triangular part of the inferior frontal gyrus: (1.85, 4.27); Right transverse temporal gyrus: (1.21, 1.60).

**Supplementary Figures**

**Supplementary Figure 1** Demographics

**
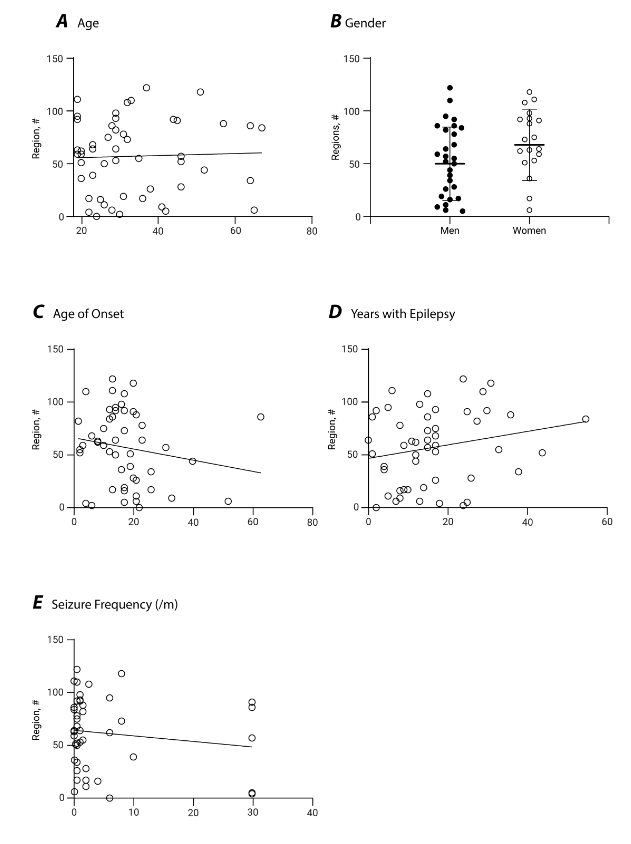
**

**Supplementary Figure 1. Demographic and clinical analyses showed no correlation to BBBD. (A)** Average number of regions with BBBD versus age (*p* = 0.8). **(B)** Average number of regions with BBBD by gender (*p* = 0.07). **(C)** Average number of regions with BBBD versus age of onset (*p* = 0.22). **(D)** Average number of regions with BBBD versus years with epilepsy (*p* = 0.14). **(E)** Average number of regions with BBBD versus seizure frequency (*p* = 0.78).

**Supplementary Figure 2** Medications


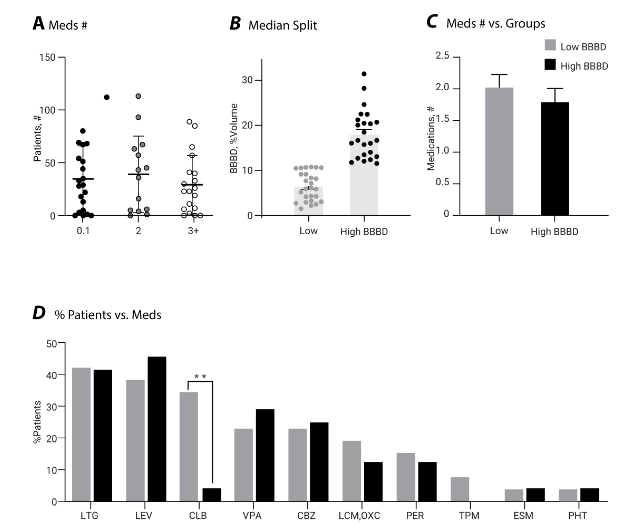


**Supplementary Figure 2. CLB has been found to be correlated with lower BBBD. (A)** Average percent of regions with BBBD by number of medications taken (*N*(0,1) = 17, *N*(2) = 14, *N*(3+) = 19. *p*(1,2 ) = 0.65, *p*(1,3) = 0.14, *p*(2,3) = 0.46). **(B)** Distribution of PWE with low and high BBBD cutoff set at the median. **(C)** Average number of medications taken by PWE with low and high BBBD. **(D)** Percent of patients who take a medicine divided into low and high BBBD groups. LTG-lamotrigine (*p* = 0.36), LEV-levetiracetam (0.63), CLB-clobazam (** 0.01), VPA-valproic acid (0.43), CBZ-carbamazepine/OXC-oxcarbazepine (0.39), LCM-lacosamide (0.2), PER-perampanel (0.87), TPM-topiramate (0.78), ESM-ethosuximide (0.78), PHT-phenytoin (0.78).

**Supplementary Tables**

**Supplementary Table 1** lesions data per PWE

| **Id** | **MRI finding** | **BBB finding** | **Clinical Diagnosis** |
| --- | --- | --- | --- |
| 1 | Focal hemosiderin, Lt. occipital & parietal | Rt. Fronto-Temporal | GTCE |
| 2 | Frontal cortical dysplasia, Lt. | Lt. Fronto-Parietal | Nocturnal frontal lobe epilepsy (NFLE) |
| 3 | White matter lesion (Rt. Superior frontal) | Rt. Hemisphere (diffuse) | Myoclonic |
| 4 | Rt. Hippocampal atrophy | Bilateral: Lt. Amygdala, Lt. Hippocampus, Rt. Frontal | Focal, unknown. |
| 5 | Old Infarct (Lt. basal nuclei) with white matter changes | Lt. Parieto-Temporal | Lt. Frontal/Temporal |
| 6 | [Heterotropia & Schizencephaly](https://www.google.com/search?sca_esv=583659685&sxsrf=AM9HkKmSZmnWnafGzipfVfNYz8FFW5KGqw:1700338409193&q=Schizencephaly&spell=1&sa=X&ved=2ahUKEwi82oKXrs6CAxUvpIkEHazDDNkQkeECKAB6BAgIEAI) Rt. Parietal | Rt. Temporo-Occipital | TLE, Susp. Lt. |
| 7 | Cortical malformation, Lt. Frontal | Lt. Frontal, Bilateral Temporal | Multi focal epilepsy Susp. Lt. frontal' |
| 8 | Post-surgical removal of cavernoma, Lt. Parietal | No BBBD | TLE, Lt. |
| 9 | Hippocampal sclerosis, Lt. | Lt. Temporal | Lt. Temporal |
| 10 | Small vessel disease, bilateral | Bilateral Diffuse | Focal epilepsy without impaired awareness. |
| 11 | Temporal lobe cavernoma, Lt. | Bilateral Fronto-Temporal | Generalized |
| 12 | Gliotic changes, post removal of AVM, Rt. Parietal | Bilateral diffuse | Frontal |
| 13 | Rt. Hippocampal sclerosis | Bilateral Temporal | TLE, Rt. |
| 14 | Rt. Hippocampal sclerosis | Bilateral Temporal. Rt. > Lt. | TLE, Rt. |
| 15 | Lt. Hippocampal atrophy | Bilateral Temporal | TLE, Lt. |
| 16 | Lt. Hippocampal sclerosis | Rt. Frontal | TLE, Rt. |
| 17 | Lt. Hippocampal sclerosis | Lt. Hippocampus | TLE, Lt. |
| 18 | Lt. temporal atrophy | Lt. Inferior frontal gyrus | TLE, Lt. |
| 19 | Rt. Hippocampal sclerosis | Rt. Frontal | TLE, Rt. |
| 20 | Lt. Hippocampal sclerosis | Lt. Fronto-temporal | TLE, Lt. |

**Supplementary Table 2** MAD per region

| **Region** | **Controls** | **Epilepsy** | **Focal Epilepsy** | **Generalized Epilepsy** | **Frontal Epilepsy** | **Temporal Epilepsy** |
| --- | --- | --- | --- | --- | --- | --- |
| **Left supramarginal gyrus** | 0.53 | 4.02 | 4.61 | 3.62 | 5.79 | 4.47 |
| **Left orbital part of the inferior frontal gyrus** | 0.64 | 3.67 | 3.9 | 3.82 | 4.49 | 3.87 |
| **Left postcentral gyrus medial segment** | 0.63 | 3.54 | 4.3 | 2.05 | 5.42 | 4.28 |
| **Left superior temporal gyrus** | 0.54 | 3.48 | 3.8 | 3.5 | 4.29 | 3.41 |
| **Right orbital part of the inferior frontal gyrus** | 0.64 | 3.46 | 3.48 | 5.58 | 3.84 | 3.63 |
| **Left frontal pole** | 0.62 | 3.4 | 3.71 | 3.16 | 4.82 | 2.13 |
| **Left opercular part of the inferior frontal gyrus** | 0.59 | 3.35 | 3.84 | 3.32 | 4.5 | 3.86 |
| **Right superior parietal lobule** | 0.66 | 3.29 | 3.63 | 3.66 | 5.16 | 2.97 |
| **Right supramarginal gyrus** | 0.48 | 3.28 | 3.53 | 4.26 | 4.7 | 3.27 |
| **Right Accumbens Area** | 1 | 3.28 | 3.3 | 3.87 | 3.2 | 3.48 |
| **Left angular gyrus** | 0.39 | 3.27 | 3.55 | 3.23 | 4.69 | 3.09 |
| **Right middle temporal gyrus** | 0.37 | 3.24 | 3.36 | 5.16 | 4.89 | 2.31 |
| **Right inferior temporal gyrus** | 0.3 | 3.24 | 3.04 | 6.16 | 4.08 | 2.5 |
| **Right inferior occipital gyrus** | 0.33 | 3.23 | 3.08 | 5.92 | 3.62 | 3.12 |
| **Cerebellar Vermal Lobules VIII-X** | 0.77 | 3.18 | 3.31 | 4.35 | 3.26 | 3.19 |
| **Right superior frontal gyrus medial segment** | 0.28 | 3.17 | 3.69 | 2.91 | 5.56 | 3.21 |
| **Right cuneus** | 0.32 | 3.13 | 3.42 | 3.75 | 4.39 | 3.04 |
| **Left middle frontal gyrus** | 0.32 | 3.12 | 3.43 | 3.3 | 4.44 | 3.22 |
| **Right middle frontal gyrus** | 0.26 | 3.09 | 3.4 | 3.93 | 4.87 | 2.78 |
| **Left superior parietal lobule** | 0.37 | 3.07 | 3.59 | 2.65 | 5.21 | 3.28 |
| **Right supplementary motor cortex** | 0.33 | 3.04 | 3.47 | 3.09 | 4.79 | 2.96 |
| **Left planum temporale** | 0.21 | 3.04 | 3.18 | 3.19 | 3.83 | 2.65 |
| **Left anterior cingulate gyrus** | 0.58 | 3.02 | 3.45 | 2.44 | 4.44 | 2.79 |
| **Right angular gyrus** | 0.48 | 3.01 | 3.14 | 4.22 | 4.72 | 2.64 |
| **Left postcentral gyrus** | 0.59 | 2.99 | 3.49 | 2.66 | 4.91 | 3.15 |
| **Right middle occipital gyrus** | 0.4 | 2.99 | 3.17 | 4.26 | 4.65 | 2.57 |
| **Left superior frontal gyrus medial segment** | 0.41 | 2.97 | 3.42 | 2.86 | 4.66 | 2.71 |
| **Right precuneus** | 0.36 | 2.95 | 3.03 | 3.37 | 3.91 | 2.79 |
| **Right postcentral gyrus medial segment** | 0.59 | 2.94 | 3.42 | 2.79 | 4.07 | 2.97 |
| **Left middle temporal gyrus** | 0.42 | 2.94 | 3.07 | 3.41 | 3.26 | 2.96 |
| **Right planum temporale** | 0.58 | 2.93 | 2.96 | 4.55 | 4.22 | 2.13 |
| **Right frontal pole** | 0.51 | 2.91 | 3.57 | 2.1 | 3.52 | 3.8 |
| **Left precentral gyrus medial segment** | 0.34 | 2.9 | 3.3 | 2.86 | 4.42 | 2.7 |
| **Left Accumbens Area** | 1 | 2.9 | 2.44 | 5.68 | 3 | 1.92 |
| **Right Cerebellum Exterior** | 0.35 | 2.89 | 2.54 | 5.97 | 3.6 | 2.08 |
| **Right Cerebral White Matter** | 0.37 | 2.88 | 2.96 | 4.26 | 4.87 | 1.9 |
| **Left parietal operculum** | 0.27 | 2.88 | 3.38 | 2.43 | 4.46 | 2.6 |
| **Right central operculum** | 0.53 | 2.88 | 2.73 | 4.95 | 3.77 | 2.01 |
| **Right transverse temporal gyrus** | 0.25 | 2.86 | 2.84 | 4.81 | 4.47 | 1.57 |
| **Right planum polare** | 0.63 | 2.86 | 2.78 | 4.65 | 3.95 | 2.09 |
| **Right precentral gyrus** | 0.46 | 2.83 | 3.04 | 3.68 | 4.4 | 2.52 |
| **Left central operculum** | 0.05 | 2.8 | 3.12 | 2.08 | 3.87 | 2.92 |
| **Right superior temporal gyrus** | 0.36 | 2.76 | 2.73 | 4.7 | 4.2 | 1.87 |
| **Left Cerebral White Matter** | 0.42 | 2.75 | 2.88 | 3.02 | 4.1 | 2.07 |
| **Right postcentral gyrus** | 0.34 | 2.74 | 3.05 | 3.44 | 4.54 | 2.54 |
| **Left temporal pole** | 0.43 | 2.73 | 2.58 | 3.33 | 3.61 | 2.1 |
| **Left precentral gyrus** | 0.41 | 2.68 | 3.14 | 2.55 | 4.59 | 2.53 |
| **Right temporal pole** | 0.69 | 2.68 | 2.5 | 4.27 | 3.99 | 1.77 |
| **Right anterior cingulate gyrus** | 0.49 | 2.67 | 3.01 | 2.23 | 4.31 | 2.65 |
| **Right opercular part of the inferior frontal gyrus** | 0.31 | 2.67 | 2.56 | 4.43 | 3.49 | 2.08 |
| **Right precentral gyrus medial segment** | 0.37 | 2.66 | 3.12 | 2.27 | 4.46 | 2.66 |
| **Right posterior orbital gyrus** | 0.3 | 2.65 | 2.54 | 4.45 | 3.99 | 1.64 |
| **Right anterior insula** | 0.63 | 2.65 | 2.51 | 4.4 | 3.65 | 1.92 |
| **Left triangular part of the inferior frontal gyrus** | 0.45 | 2.64 | 2.99 | 2.15 | 3.73 | 2.93 |
| **Right occipital fusiform gyrus** | 0.33 | 2.6 | 2.06 | 5.42 | 2.71 | 1.76 |
| **Left planum polare** | 0.71 | 2.59 | 2.7 | 3.05 | 2.29 | 2.95 |
| **Right Amygdala** | 0.67 | 2.57 | 2.62 | 3.42 | 4.63 | 1.42 |
| **Right Caudate** | 0.51 | 2.57 | 2.79 | 2.96 | 4.08 | 1.99 |
| **Right superior occipital gyrus** | 0.43 | 2.57 | 2.78 | 3.38 | 3.99 | 2.24 |
| **Left precuneus** | 0.36 | 2.55 | 2.75 | 2.47 | 3.32 | 2.46 |
| **Right anterior orbital gyrus** | 0.56 | 2.54 | 2.67 | 2.55 | 4.4 | 1.13 |
| **Left lateral orbital gyrus** | 0.51 | 2.52 | 2.56 | 2.17 | 4.22 | 1.43 |
| **Left middle occipital gyrus** | 0.23 | 2.51 | 2.87 | 2.15 | 3.96 | 2.55 |
| **Left inferior occipital gyrus** | 0.49 | 2.49 | 2.68 | 3.29 | 3.01 | 2.65 |
| **Right posterior insula** | 0.41 | 2.45 | 2.22 | 4.88 | 3.55 | 1.3 |
| **Left transverse temporal gyrus** | 0.29 | 2.42 | 2.31 | 2.58 | 2.63 | 1.96 |
| **Left superior occipital gyrus** | 0.23 | 2.4 | 2.74 | 2.24 | 3.59 | 2.85 |
| **Right superior frontal gyrus** | 0.34 | 2.39 | 2.57 | 3.39 | 4.38 | 1.78 |
| **Left medial frontal cortex** | 0.52 | 2.39 | 3.02 | 1.39 | 3.3 | 2.51 |
| **Left calcarine cortex** | 0.44 | 2.39 | 2.6 | 2.77 | 3.11 | 2.36 |
| **Right medial frontal cortex** | 0.48 | 2.38 | 2.77 | 2.25 | 3.74 | 2.03 |
| **Cerebellar Vermal Lobules VI-VII** | 0.62 | 2.37 | 2.41 | 3.59 | 2.48 | 2.31 |
| **Right Basal Forebrain** | 0.34 | 2.35 | 2.74 | 2.35 | 3.01 | 2.88 |
| **Left Cerebellum White Matter** | 0.6 | 2.34 | 2.23 | 3.39 | 3.1 | 1.59 |
| **Left medial orbital gyrus** | 0.39 | 2.33 | 2.38 | 2.84 | 2.55 | 1.99 |
| **Right entorhinal area** | 0.35 | 2.32 | 2.49 | 3.2 | 4.38 | 1.66 |
| **Right medial orbital gyrus** | 0.67 | 2.31 | 2.35 | 3.26 | 3.04 | 1.77 |
| **Left superior frontal gyrus** | 0.59 | 2.3 | 2.52 | 2.04 | 4.21 | 2.01 |
| **Right Putamen** | 0.62 | 2.3 | 2.04 | 4.54 | 3.16 | 1.28 |
| **Right lateral orbital gyrus** | 0.62 | 2.26 | 2.18 | 2.97 | 2.82 | 1.55 |
| **Left Basal Forebrain** | 0.39 | 2.26 | 2.18 | 3.43 | 2.06 | 2.17 |
| **Right parietal operculum** | 0.44 | 2.22 | 2.13 | 3.53 | 3.11 | 1.46 |
| **Left supplementary motor cortex** | 0.44 | 2.22 | 2.38 | 2.38 | 3.02 | 2.11 |
| **Left Cerebellum Exterior** | 0.33 | 2.22 | 2.37 | 2.98 | 3 | 2.1 |
| **Left anterior orbital gyrus** | 0.41 | 2.21 | 2.04 | 1.97 | 2.92 | 1.45 |
| **Right lingual gyrus** | 0.24 | 2.21 | 1.9 | 4.57 | 2.23 | 1.67 |
| **Left cuneus** | 0.15 | 2.2 | 2.36 | 2.11 | 3.31 | 1.93 |
| **Right fusiform gyrus** | 0.03 | 2.19 | 1.8 | 5.01 | 3.45 | 0.63 |
| **Right Cerebellum White Matter** | 0.64 | 2.19 | 1.94 | 4.43 | 2.99 | 1.28 |
| **Left posterior orbital gyrus** | 0.43 | 2.16 | 2.33 | 2.58 | 3.11 | 1.72 |
| **Left inferior temporal gyrus** | 0.5 | 2.16 | 2.11 | 2.93 | 2.52 | 1.92 |
| **Right parahippocampal gyrus** | 0.24 | 2.14 | 1.94 | 4.28 | 3.29 | 1.13 |
| **Left posterior cingulate gyrus** | 0.6 | 2.14 | 2.27 | 2.03 | 2.69 | 2.06 |
| **Left Caudate** | 0.58 | 2.07 | 1.91 | 3.54 | 2.48 | 1.69 |
| **Right middle cingulate gyrus** | 0.52 | 2.05 | 2.49 | 1.35 | 3.01 | 2.22 |
| **Left entorhinal area** | 0.66 | 2.02 | 2.06 | 2.15 | 2.73 | 1.62 |
| **Left Amygdala** | 0.68 | 2.02 | 2.01 | 2.5 | 2 | 1.89 |
| **Right Thalamus Proper** | 0.44 | 1.97 | 1.81 | 3.61 | 2.62 | 1.27 |
| **Left Thalamus Proper** | 0.47 | 1.96 | 1.76 | 3.04 | 2.22 | 1.33 |
| **Right triangular part of the inferior frontal gyrus** | 0.59 | 1.94 | 2.04 | 2.34 | 3.04 | 1.5 |
| **Right Hippocampus** | 0.55 | 1.94 | 1.76 | 3.75 | 2.53 | 1.23 |
| **Left frontal operculum** | 0.41 | 1.91 | 2.1 | 1.5 | 2.73 | 2.48 |
| **Right calcarine cortex** | 0.34 | 1.91 | 1.8 | 3.32 | 2.67 | 1.19 |
| **Right frontal operculum** | 0.46 | 1.87 | 1.58 | 3.18 | 2.34 | 1.41 |
| **Right posterior cingulate gyrus** | 0.43 | 1.85 | 1.91 | 1.87 | 2.46 | 1.63 |
| **Left occipital fusiform gyrus** | 0.25 | 1.85 | 1.81 | 2.62 | 1.09 | 2.26 |
| **Left lingual gyrus** | 0.42 | 1.84 | 1.49 | 3.8 | 1.17 | 1.71 |
| **Left gyrus rectus** | 0.68 | 1.83 | 2.07 | 1.82 | 2.62 | 2.18 |
| **Right gyrus rectus** | 0.59 | 1.8 | 1.87 | 2.39 | 2.66 | 1.78 |
| **Right Ventral DC** | 0.64 | 1.8 | 1.67 | 3.2 | 2.51 | 1.07 |
| **Left Ventral DC** | 0.5 | 1.8 | 1.86 | 2.16 | 1.99 | 1.22 |
| **Left posterior insula** | 0.42 | 1.73 | 1.73 | 1.85 | 2.29 | 1.39 |
| **Left Putamen** | 0.5 | 1.69 | 1.48 | 2.32 | 2.23 | 0.78 |
| **Left parahippocampal gyrus** | 0.35 | 1.69 | 1.83 | 1.95 | 2.15 | 1.28 |
| **Left anterior insula** | -0.02 | 1.67 | 1.76 | 2.13 | 2.09 | 1.7 |
| **Left Hippocampus** | 0.5 | 1.63 | 1.62 | 2.07 | 1.59 | 1.39 |
| **Left middle cingulate gyrus** | 0.34 | 1.62 | 1.97 | 1.17 | 2.39 | 1.78 |
| **Cerebellar Vermal Lobules I-V** | 0.59 | 1.62 | 1.61 | 2.15 | 1.86 | 1.33 |
| **Left subcallosal area** | 0.41 | 1.47 | 1.57 | 1.44 | 1.6 | 1.19 |
| **Brain Stem** | 0.31 | 1.38 | 1.21 | 2.42 | 1.96 | 0.8 |
| **Left fusiform gyrus** | 0.43 | 1.34 | 1.34 | 1.38 | 1.64 | 0.96 |
| **Right subcallosal area** | 0.72 | 1.3 | 1.42 | 1.41 | 1.79 | 1.07 |
| **Left occipital pole** | 0.37 | 1.26 | 1.37 | 1.35 | 1.6 | 1.76 |
| **Right occipital pole** | 0.41 | 0.83 | 0.92 | 1.25 | 0.9 | 1.2 |

**Supplementary Table 3** Medications, high and low BBBD

| **Medication** | **% Patients, Low BBBD** | **% Patients, High BBBD** |
| --- | --- | --- |
| Sodium Valproate | 20 | 30 |
| Carbamazepine | 10 | 23.33 |
| Levetiracetam | 30 | 36.67 |
| Lamotrigine | 50 | 36.67 |
| Lacosamide | 25 | 10 |
| Brivaracetam | 20 | 6.67 |
| Oxcarbazepine | 10 | 3.33 |
| Ethosuximide | 5 | 3.33 |
| Topiramate | 5 | 3.33 |
| Clobazam | 40 | 6.67 |
| Perampanel | 15 | 13.33 |
| Phenytoin | 5 | 3.33 |
